# Geospatial modeling of land cover change in the Chocó-Darien global ecoregion of South America; One of most biodiverse and rainy areas in the world

**DOI:** 10.1371/journal.pone.0211324

**Published:** 2019-02-01

**Authors:** J. Camilo Fagua, R. Douglas Ramsey

**Affiliations:** 1 RS/GIS Laboratory, Department of Wildland Resources and the Ecology Center, Utah State University, Logan, Utah United States of America; 2 CIAF, Instituto Geográfico Agustín Codazzi, Bogotá DC, Colombia; 3 School of Informatics, Computing, and Cyber Systems, Northern Arizona University, Flagstaff, Arizona, United States of America; Oregon State University, UNITED STATES

## Abstract

The tropical rain forests of northwest South America fall within the Chocó-Darien Global Ecoregion (CGE). The CGE is one of 25 global biodiversity hotspots prioritized for conservation due to its high biodiversity and endemism as well as threats due to deforestation. The analysis of land-use and land-cover (LULC) change within the CGE using remotely sensed imagery is challenging because this area is considered to be one of the rainiest places on the planet (hence high frequency of cloud cover). Furthermore, the availability of high-resolution remotely sensed data is low for developing countries before 2015. Using the Random Forest ensemble learning classification tree system, we developed annual LULC maps in the CGE from 2002 to 2015 using a time series of cloud-free MODIS vegetation index products. The MODIS imagery was processed through a Gaussian weighted filter to further correct for cloud pollution and matched to visual interpretations of land cover and land use from available high spatial resolution imagery (WorldView-2, Quick Bird, Ikonos and GeoEye-1). Validation of LULC maps resulted in a Kappa of 0.87 (Sd = 0.008). We detected a gradual replacement of forested areas with agriculture (mainly grassland planted to support livestock grazing), and secondary vegetation (agriculture reverting to forest) across the CGE. Forest loss was higher between 2010–2015 when compared to 2002–2010. LULC change trends, deforestation drivers, and reforestation transitions varied according to administrative organization (countries: Panamanian CGE, Colombian CGE, and Ecuadorian CGE).

## Introduction

Land-use and land-cover (LULC) change brought about by human development are constantly reshaping natural regions at local, national to global scales [1–4]. Evaluating these landscape level changes annually within regions where the natural condition is composed of tropical rain forests is difficult due to the high amounts of cloud cover obscuring remote sensing instruments. Globally, these regions are suffering significant LULC change [2,5,6] causing much concern due to its potential effect on climatic change, biodiversity loss, hydrologic alteration, soil degradation, and loss of ecosystem services [3,7]. Some national and global estimates have found that deforestation due to LULC change was significantly higher than reforestation in Central and South America [2,6,8,9]. Conversely, other LULC change studies in the same region show a reforestation trend during similar time periods [5,10]. Although the methodologies were different, these contradictory results suggest that LULC change could be highly heterogeneous in time and space in the tropical rain forest domain. It also shows that consistent and accurate information about the LULC dynamic is critical for the management and protection of these forests.

**Fig 1:**
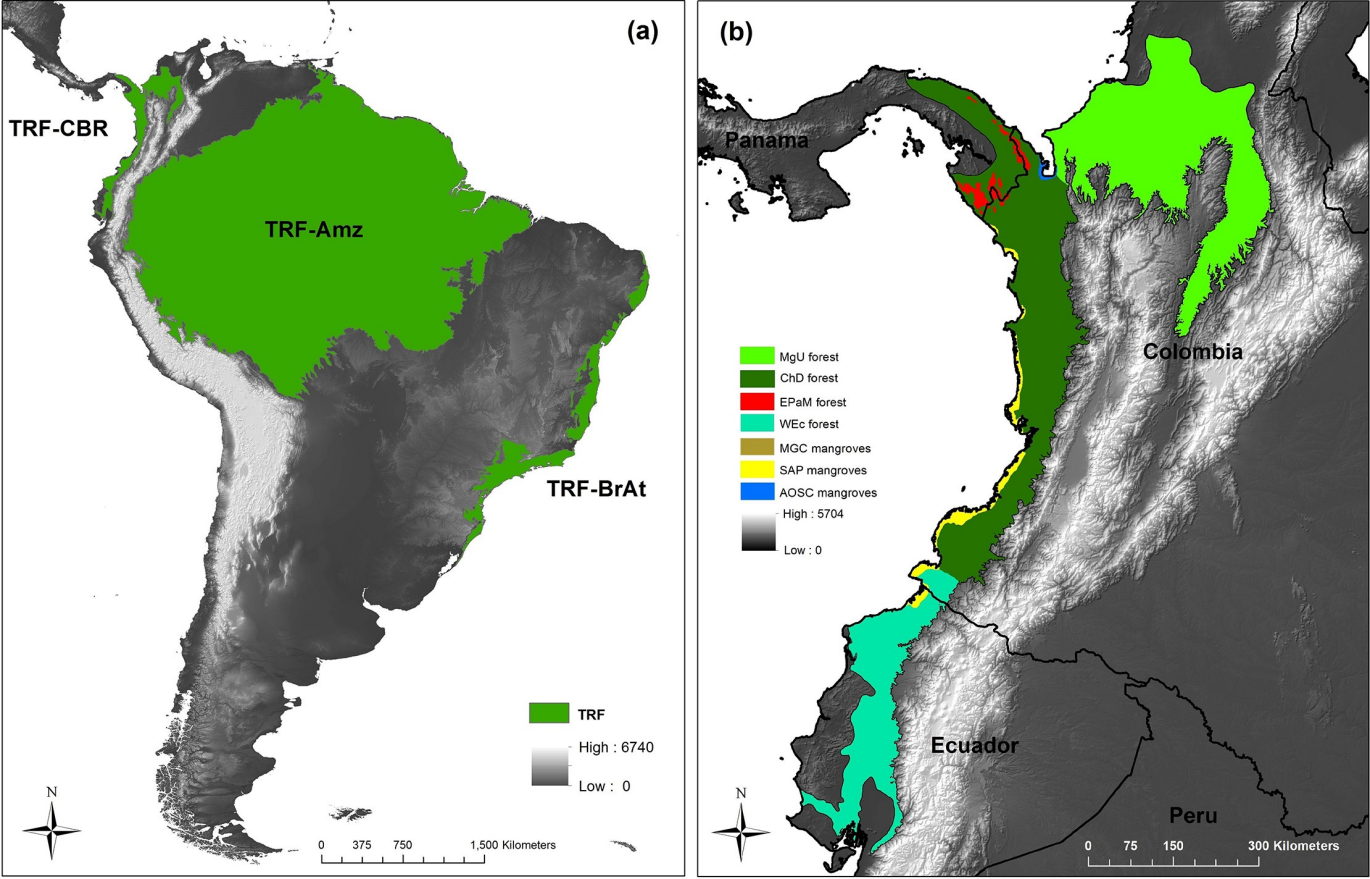
The Chocó-Darien Global Ecoregion (CGE): (a) estimated historical extent of Tropical Rain Forest (TRF) in South America: TRF-CGE (estimated TRF in CGE), TRF-Amz (estimated TRF in Amazon basin), and TRF-BrAt (estimated TRF in Brazilian Atlantic Forest). (b) The Chocó-Darien Global Ecoregion (CGE): This global ecoregion is formed by three smaller ecoregions: Magdalena-Urabá Moist forests (MgU), Chocó-Darién Moist Forests (ChD), and Western Ecuador Moist Forests (WEc). Other four ecoregions have small sections in the CGE: Eastern Panamanian Montane Forests (EPaM), South American Pacific (SAP) Mangrove, Amazon-Orinoco-Southern Caribbean (AOSC) Mangrove, and Mesoamerican Gulf-Caribbean (MGC) Mangrove.

Tropical rain forests are currently the most biodiverse landscapes on our planet [11–13]. In South America, tropical rain forests form three well define natural regions; the Amazon Basin, the Brazilian Atlantic Forest, and the Chocó-Darien Global Ecoregion (CGE; also known as the Chocó Biogeographic Region) (Fig 1A). The CGE is a lowland area located along the pacific coast of eastern Panamá, western Colombia, and northwestern Ecuador and has been declared as one of the top 25 global hotspots for conservation priorities (Fig 1B) [4,12–14]. Historically, most of the effort to estimate forest cover and the LULC dynamic have been focused on the Amazon Basin, the largest tropical rain forests in the world [1,15–21]. Likewise, LULC dynamic in the Brazilian Atlantic Forest has been well studied [16,22–24]. Despite the fact that the CGE is recognized as one of the world's most biologically diverse regions [4,25], it has not received the same level of study relative to its LULC dynamic. The countries that share the CGE and their research organizations have conducted studies of the CGE within their own boundaries [8,10,26], but these studies have used different methodologies and/or sensors, and do not allow for valid comparisons to evaluate the region as a whole. Furthermore, regional and global studies of LULC change have been done for large areas that include the CGE [3,5,27,28]. However, these analyses have used general LULC categories, restricting the identification of deforestation drivers occurring in the CGE and giving only a general idea about the region’s LULC dynamics. A study of LULC dynamics focused specifically on the CGE is a fundamental need to guide proper management and conservation for this area.

Past LULC change studies in neotropical rain forest regions have focused on the gain and/or loss of forest cover [2,5,8,10,26]. Local studies within the Amazon Basin and Brazilian Atlantic Forest ecoregions have accurately identified direct drivers of deforestation and their temporal and spatial variation [18,29–31]. However, within the CGE, much less is known about the direct drivers of deforestation. The United Nations Framework Convention on Climate Change negotiations has encouraged developing countries to spatially map direct drivers of deforestation [32,33]. While some studies have shown reforestation trends due to apparent abandonment of agriculture lands, this reforestation process has only been slightly studied in the Colombian CGE [34].

An analysis of LULC change across the CGE is a challenge when remote sensing imagery is used because this area is considered one of the rainiest on the planet with an annual average rainfall between 8000 to 13000 mm [35]. Furthermore, the availability of high spatial resolution remote sensing data before 2015 is low for Panamá, Colombia and Ecuador. Consequently, the satellite images that are available (Landsat, Satellite Pour l'Observation de la Terre (SPOT), and RapidEye, for example) usually have a high percentage of cloud cover, making it difficult for the development of regional land cover maps.

Nevertheless, over the past decade, methodologies using MODIS (MODerate resolution Imaging Spectroradiometer) data have generated standard periodic cloud-free products aimed at monitoring vegetation across the globe [36,37]. Merging these MODIS products with available high spatial resolution (e.g. WorldView, Ikonos, QuickBird, GeoEye) imagery, used as a reference data source, with learning algorithms (e.g. Random Forest) offers potential for studying region-wide LULC in areas like the CGE. These standardized remote sensing products can also support biodiversity monitoring by coupling with essential biodiversity variables (variables that quantify biodiversity changes over time and across space), opening new possibilities for conservation efforts in tropical areas similar to the CGE [38–40].We have applied a combination of methodologies developed by various authors to multi-temporal MODIS imagery to generate yearly LULC maps across the CGE from 2002 to 2015. Our aim was to analyze LULC temporal dynamics across this ecoregion and address the following objectives: 1) Evaluate LULC change trends in the CGE and determine its heterogeneity in time and space. 2) Spatially identify deforestation drivers, reforestation transitions (cover types that represent secondary forest-like vegetation), and quantify their change in time and space. We discuss the types of information that are useful for conservation of biodiversity in the CGE relative to its administrative organization (countries).

## Materials and methods

### Study area

The Chocó-Darien Global Ecoregion (CGE) is a lowland area located along the Pacific coast from Panamá, through Colombia, and into northwestern Ecuador and includes the lowland of the Magdalena river valley (Fig 1B) [4,13,14]. The CGE became separated from the Amazon tropical rain forest by the uplift of the Andes beginning around 25 million years ago. As a consequence, groups of endemic species emerged producing a significant impulse of diversification. Another substantial array of new species arose because of the relatively recent formation of the Isthmus of Panamá (three million years ago), an geological event that formed a land bridge for plants and animals from North and South America [13,41]. Presently, it is estimated that the CGE has about 11,000 species of vascular plants (2,250 endemics), 900 species of birds, 350 species of amphibians (210 endemics), and 210 species of reptiles (63 endemic) [12,14]. It was estimated in the year 2000 that the remaining tropical rain forest within the CGE covers approximately 24% of its original distribution [12]. Due to this level of deforestation and the high number of endemic species, the CGE was declared as one of the 25 global hotspots for conservation priorities [4]. According to the World Wildlife Fund (2016), the CGE is formed by four smaller terrestrial ecoregions: Chocó-Darién Moist Forests (73028.6 km^2^), Magdalena-Urabá Moist Forests (76396 km^2^), Western Ecuador Moist Forests (33861.1 km^2^), and smaller sections of Eastern Panamanian Montane Forests (2632.4 km^2^). Also, sections of three mangrove ecoregions are found along the CGE coast: South American Pacific Mangrove (6252.4 km^2^), Amazon-Orinoco-Southern Caribbean Mangrove (702.9 km^2^), and a small area of Mesoamerican Gulf-Caribbean Mangrove (50.2 km^2^) (Fig 1B). We did not include mangrove ecoregions in our LULC change analysis because they are small areas of marine wetlands (3% of the CGE) and our study was focused on terrestrial tropical rain forest.

### LULC maps

We generated a temporal set of LULC maps based on a Random Forest [42] classification in which we modeled a categorical response variable that identified eight LULC classes. Random Forest is an ensemble learning algorithm that constructs multiple classification trees (e.g. 500 individual trees) by bootstrapping samples from an input data set, and combines the predictions from all the trees to identify a modal response [43]. Random Forest is one of the most robust statistically-based classification techniques and presents two main advantages for our analysis; it has low sensitivity to the overfit produced by collinearity among predictors [44] and allows for use of different types of response and predictor variables (e.g. numerical, binary, categorical) in the classification process [45].

The mapping of these LULC classes was accomplished by training MODIS cloud-free temporal image mosaics using 22 sampling sub-regions covering 20,708.6 km^2^ of total land area within the CGE. These 22 sampling sub-regions corresponded with locations of available high-resolution imagery. The cloud-free MODIS vegetation index products MOD13Q1.V006 (tiles h10v07, h10v08, h10v09, and h09v09) were downloaded from the NASA Distributed Active Archive Center and processed to transform the standard sinusoidal projection to WGS84 geographic coordinate system. This transformation resulted in a calculated pixel size of 231.3 m^2^.

### Response variable (land-cover/land-use classes)

Training samples for each LULC class were collected by visually interpreting four types of high spatial resolution images: WorldView-2, Ikonos, Quick Bird, and GeoEye-1. To improve visual interpretation of LULC classes, the multispectral bands from these sensors were fused to their corresponding panchromatic band (S1 Table). We reviewed previous regional LULC studies within the CGE to help define our LULC classes [8,26,28,34]. From these studies, we established eight general LULC classes.

1) Woody vegetation: This type of vegetation included tropical rain forest with trees taller than 30 m, secondary vegetation (shrubs and smaller trees) as well as mosaics of both. This is the primary natural cover type that occurs within the CGE [13,46,47]. Initially, forest and secondary vegetation were established as two different LULC classes; however, the Random Forest classification could not adequately separate them. Likewise, we created a mixed woody class (pixels with 20%–80% of woody and the rest the pixel cover by agricultural land), but the Random Forest classification could not separate this cover type either. Consequently, after doing a Fuzzy accuracy analysis [48] of a preliminary classification, forest and shrub were merged into a woody vegetation class. 2) Wetland: The CGE has a complex of river basins with swamps and shallow lakes ("ciénagas") covering large areas along the rivers. Wetland areas were absent in previous LULC work performed within portions of the CGE [5,8,10,49] and as a result have been markedly underestimated in global maps [2,28]. 3) Grassland: Introduced grass species which are used primarily for cattle grazing [50]. Within the CGE, large areas of native grasses do not occur as natural vegetation [46,47]. 4) Crops: Agriculture consisting of annual or semiannual crops (corn, sugar canna, plantain, mainly). 5) Palm plantations: Extensive areas of the CGE have been cultivated with African palm (*Elaeis Guineensis* Jacq) [51]. These palms take about three years to mature and produce oil. The useful life of a palm plantation is about 25 years at which point plantations are replanted with younger palms [52,53]. In terms of remote sensing, this relatively stable structure of palm plantations allowed its identification as a LULC class using our imagery resources. 6) Settlements and infrastructure. 7) Continental water including rivers and lakes. 8) Bare areas: This class was not taken into account in the final analysis due to its low representation.

The 192,924 km^2^ of land corresponding to the CGE was divided into square sample areas of 231.3 m x 231.3 m to match the MODIS pixel size. Based on this grid, a stratified sampling was applied to the area intersecting the aforementioned high spatial resolution images as follows: we visually identified sample squares with 100% of any of the eight LULC categories. We then superimposed a second grid of 1 km^2^ as spatial filter to select one square of 231.3 m^2^ for every 1 km^2^ square. This spatial filter ensured that sample sites were separated by 693 m or more. By doing this, we identified 18,559 sample sites classified as one of the eight LULC classes. To estimate the error rate for the visual interpretation, we compared our visual interpretation with the visual interpretations of the Corine Land cover project for Colombia [54], which used many resources (high spatial resolution imagery, aerial photos, Landsat, and field visits) to reach the best possible visual interpretation of land cover. We coupled 375 of our MODIS sampling sites to the corresponding interpretations from the Corine Land Cover effort for the years 2002, 2003, and 2007. The agreement between both interpretations resulted in a kappa of 0.93 (Accuracy = 0.95), showing a high level of consistency between both interpretations.

### Predictor variables

**Fig 2:**
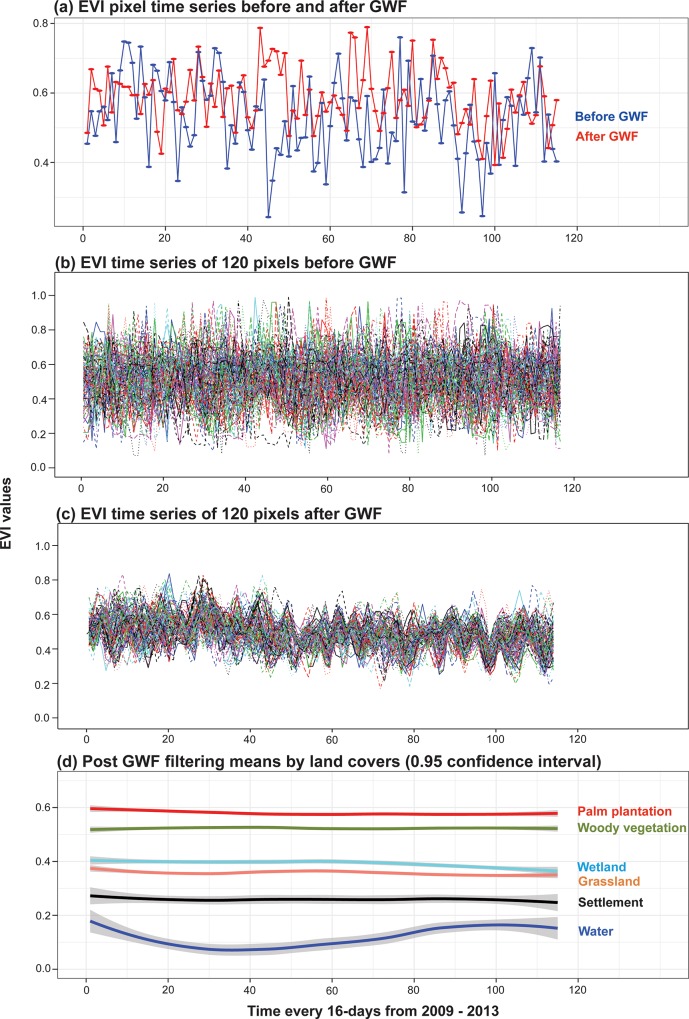
An example of the time series filtering procedure from 2009 to 2013 using the Gaussian weighted filter (GWF). GWF improved the identification of land cover using the MODIS bands and indices. (a) A time series EVI pixel of woody vegetation before and after filtering; outliers are replaced with estimates calculated by the Gaussian weighted filter. (b) Temporal variation of 120 pixels corresponding to woody vegetation before filtering and (c) the same 120 pixels after filtering; the variance of these 120-time series is reduced. (d) Post filtering results–simplified to means, for 120 woody vegetation pixels, 116 grassland pixels, 542 palm plantation pixels, 99 settlement pixels, 101 water pixels, and 454 wetland pixels. GWF increased the differentiation among these land covers. The GWFs were applied using the Terrset software [61] with a temporal filter length of 5.

Five MODIS-based predictor variables were generated from the MOD13Q1 product (16-Day L3 Global 250 m Vegetation Indices). The MOD13Q1 product provides the highest quality pixels from 16 daily images for four spectral bands: blue (459 nm -479 nm), red (620 nm –670 nm), near infrared (NIR: 841 nm– 876 nm), and mid-infrared (MIR: 2105 nm –2155 nm); as well as two indices: Enhanced Vegetation Index (EVI) and the Normalized Difference Vegetation Index (NDVI). EVI reduces atmospheric influences on vegetation detection and improves identification of vegetation with dense canopies, such as tropical forest, where NDVI tended to saturate [55]. However, we used both EVI and NDVI as predictors because NDVI has been equivalent or better than EVI detecting vegetation covers with low biomass and canopies, such as grassland, shrub, crop, and subtropical deciduous forests [56–59]. The MOD13Q1 product also provides layers that estimate vegetation index quality, sensor view zenith, solar zenith angles, individual pixel Julian day, and a pixel reliability ranking. For our analysis we did not use the blue spectral band due to its lower spatial resolution, 462.7 m^2^. The yearly collection of MOD13Q1 data consists of 23 temporally sequential periods (365 days and 16 days per period) for every year from 2001 to the present. We utilized the entire range of data from 2001 through the end of 2015, for a total of 345 individual measurements of red, NIR, MIR, NDVI and EVI for every 231.3 meter pixel in the CGE. Although the MOD13Q1 product attempts to evaluate pixel quality as a function of radiometric and atmospheric conditions (cloud interference), these data can still contain anomalies that are caused by factors not relevant to the amount of photosynthetically active surface cover, namely atmospheric conditions. To account for these anomalies and therefore the uncertainty within the vegetation index products, we applied a Gaussian weighted filter to the 23 temporal periods for each year and for each of the five spectral variables. This filter reduced the variation of the MODIS bands and indices and replaced outlier values with estimates calculated by the Gaussian weighted series (Fig 2). We used the output of the Gaussian weighted filter to estimate an annual mean for each band and index, and we used these means as predictor variables [37,60]. This analysis was performed using TerrSet Geospatial Monitoring and Modeling Software from Clark Labs [61], with each year from 2001 to 2015 representing a time series cycle (a total of 15 time series cycles) with a temporal filter length or “window” of 5. In addition to the MODIS-based predictor variables, we included the SRTM90 (NASA Shuttle Radar Topographic Mission) elevation data and its corresponding slope values as ancillary data in support of the image classification process [62,63]. Elevation and slope have been found to affect the type of land cover that occurs in a specific area; forest tends to be preserved in places with higher altitude and slope (due to a more difficult access) while crops and palm plantations occur in places with low slope [52,64]. As well, the wetlands in the CGE are located in areas with an altitude near or under the sea level [47,65]. Additionally, the elevation data of SRTM90 could be affected by densely vegetated areas [66–69] and wetlands [70]. SRTM has a spatial resolution of 90 m^2^ and was, therefore, resampled to 231.35 m^2^ to match the MOD13Q1 pixels using a bilinear interpolation. From this resampled digital elevation model, SRTM elevation and topographic slope were extracted for each training side.

### Random forest classification

A total of 18,559 training sites located within the 22 sampling areas were classified visually into our eight LULC classes using high resolution imagery. The training site database, therefore, contained the interpreted LULC class as well as the predictor variables of temporal spectral and vegetation index values (NDVI and EVI) for each year along with SRTM values and topographic slope where columns represented the response and predictor variables and rows consisted of the 18,559 observations (S2 Table). The Random Forest algorithm operates by constructing a large number of decision trees from random subsets of predictor variables and the resulting classification consists of the modal response of all trees for a particular outcome [45,71]. We used the R statistical packages ‘‘randomForest’ [43] and “ModelMap” [71] to generate our yearly LULC maps (the R code to build a map is available in S1 Codes). The randomForest utility in R generated a default number of trees (500), using a 80% subset of the samples (training subset) for every bootstrap iteration and the square root of the number of predictors as the number of predictors used to identify a split at each node. This RF model can provide accuracy estimates using OOB (or Out-of-Bag, a first independent subset from the training data) [45] or using a second independent group formed by the 20% of samples that were not used as training subset (testing subset). We reported the kappa from the second independent group to reduce a possible overestimation in the accuracy [72]. Accuracy estimates included Kappa (K), which was categorized into the following ranges of agreement: poor K < 0.4, good K 0.4 < K< 0.75, excellent K > 0.7575 [73], as well as percent omission and commission errors for each LULC class.

A Random Forest classification is accomplished using available training data and therefore is subject to training data distribution amongst the different response classes. The dominant land cover category in our study area consisted of the woody vegetation class. Of our 18,559 sample sites, 14,228 samples (76%) consisted of the woody vegetation class. In order to detect the potential impact of this large sample size relative to other land cover categories on the accuracy of minority classes, we randomly reduced the samples representing the woody vegetation class from 14,228 to 1,144 to match the sample size of the second most prevalent class, Grassland [74] (S3 Table). Random Forest classifications were run on both sample sets and the validation results (using a second independent group) showed that K for the original data was 0.872 and K for the reduced samples of woody vegetation class was 0.876, commission and omission errors were similar for both sample distributions (S4 and S5 Tables). Consequently, we decided to use the original sample set of 18,559 training samples. Using this methodology, we developed a RF-based LULC classification for each of the 15 years using SRTM values, topographic slope, combined with MOD13Q1 MODIS data for each year as predictors for that year. We used the R package ‘ModelMap’ which uses the ‘RGDAL’ libraries to generate LULC maps using the RF model outputs. For all of the 15 individual years, our LULC maps reached a high accuracy of K = 0.872, with a standard deviation of 0.008.

### Woody vegetation split

As we describe before, woody vegetation could not be separated into forest and shrub (secondary vegetation) classes. These two LULC classes had similar spectral and NDVI—EVI signatures provided by the MODIS data and consequently, Random Forest classification could not separate them. This spectral similarity is common in tropical rain forests when using other multi-spectral sensors such as Landsat [75]. For that reason, a final refinement after the RF classifications was applied to the annual LULC maps generated from the annual sequence of MODIS imagery. Pixels classified as woody vegetation were converted to forest when that pixel was classified as woody vegetation for every year of our sequence (2001–2015). On the other hand, if a pixel was classified as woody vegetation on the last year of our sequence (2015), but in previous years that pixel was classified as another type such as grassland, crop, or palm plantation, it was recoded as secondary vegetation. A similar methodology had been applied previously in Amazonian forests [1]; this seemed like a logical method of splitting the woody vegetation class into forest and secondary vegetation since the year-to-year accuracy of each LULC map was high and the time required for succession to reach the canopy structure of a mature forest in other neotropical rain forest ranges between 190–217 years [76,77]. Likewise, shrubby vegetation typically takes over 20 years in TRF areas to develop arborescent structures [78–80]. In other words, it is improbable that forests converted to a farm-like land-use will reach a forest-like stage in 15 years or less. Consequently, these pixels were considered secondary vegetation (landscapes converting from a farm-based land-use to natural vegetation). Based on this logic, we developed 14 final LULC maps (2002 to 2015) that included eight LULC classes: forest, secondary vegetation, wetland, grassland, crops, palm plantations, settlement, and continental water. The time series maps started in 2002 due to our methodology for splitting woody vegetation needs an initial sequence of annual maps, 2001–2002. To test accuracy of the secondary vegetation class, 191 pixels mapped as secondary vegetation were randomly selected and independently classified using visual interpretation of the available high resolution images. The accuracy of our secondary vegetation classification averaged 84.2% with a standard deviation across the years of 10.4.

### Analysis of LULC change

#### LULC change trends

To determine LULC change trends, we estimated non-parametric Pearson correlations between the area occupied for every LULC class and the corresponding year in the annual sequence. A significant positive Pearson’s correlation coefficient indicates a significant increase in the trend of that specific LULC class while a negative correlation coefficient indicates a significant reduction in area as years progress. This methodology has been applied previously to other LULC analyses [10,81]. Pearson correlations were estimated at two spatial levels: 1) the entire CGE, and 2) the areas of the CGE corresponding to the countries that share this global ecoregion (Panamá, Colombia and Ecuador). Pearson correlations also were calculated in two time periods for each of the two spatial levels: from 2002 to 2010 and from 2010 to 2015. We chose these two time periods because we found that woody vegetation, forest, and secondary vegetation trends (main objectives of our analysis) significantly changed around 2010. Additionally, other studies that include the CGE analyzed LULC change from 2001 to 2010 [5,8–10]: therefore, an analysis of temporal change between 2002–2010 provided an opportunity to compare our results with other studies.

#### Drivers of deforestation and farm conversion to secondary vegetation

To quantify the direct drivers of deforestation (direct drivers refer to the land cover that replaces forest cover) [82,83], we identified the following transitions. (1) Deforestation due to cattle grazing operations as indicated by areas of forest or secondary vegetation replaced by grassland. (2) Deforestation by annual or semiannual crops as indicated by areas of forest and secondary vegetation replaced by crops. (3) Deforestation by extensive palm plantations as indicated by areas of forest and secondary vegetation replaced by palm. (4) Deforestation by infrastructure and urban expansion as indicated by areas of forest and secondary vegetation replaced by human development. Conversely, we quantified the conversion from every farming land-use (grassland, crop, and palm plantations) to secondary vegetation as reforestation transitions. These deforestation and reforestation transitions were estimated for two temporal sequences 2002–2010 and 2010 2015 (using the first year of every sequence as the base year).

## Results

**Fig 3:**
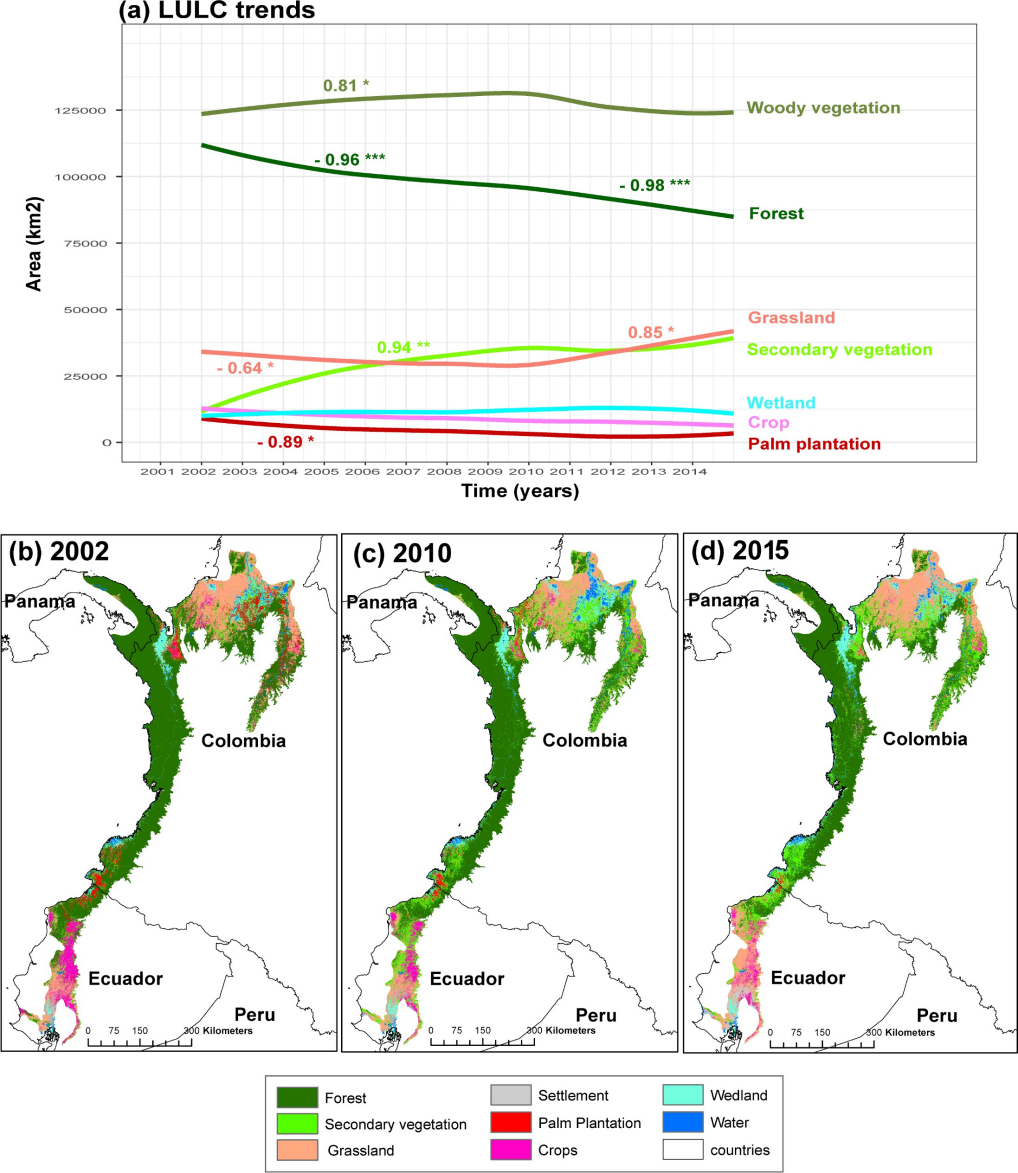
Land-use and land-cover (LULC) change trends in The Chocó-Darien Global Ecoregion (CGE). Significant correlation coefficients (R) are shown for the two temporal periods 2002–2010 and 2010–2015 (a). Significant P range values; P<0.001(***), P<0.01(**), and P<0.05(*). LULC maps for 2002, 2010 and 2015 are showed (b, c, d).

In 2002, 63.9% of the CGE (120,246 km^2^) was classified as woody vegetation (forest and secondary vegetation combined). In 2010, woody vegetation increased to 68.5% (128,801.8 km^2^), and in 2015, 65.5% (123,320.6 km^2^). In other words, woody vegetation increased 4.6% between 2002–2010 and reduced 3% between 2010–2015. For woody vegetation, 90.4% was identified as forest in 2002, 72.1% in 2010 and 67.6% in 2015. LULC trends for the entire CGE shows that secondary vegetation increased significantly from 2002 to 2010 (R = 0.94, p< 0.01) whereas forest (R = -0.96, p< 0.001) and agriculture (R = -0.64, p< 0.05 for grassland; R = -0.64, p<0.06 for crop; R = -0.89, p< 0.02 for palm) decreased showing a progressive replacement of forest and agriculture with secondary vegetation (Fig 3). Some of these trends changed between 2010 to 2015; woody vegetation declined but not significantly, forest maintained its decreasing trend (R = -0.98, p< 0.001), and grassland increased (R = 0.85, p< 0.02) while the other agricultural land use trends did not show significant trends (Fig 3). These results show that deforestation transitions (changes from forest or secondary vegetation to farm covers) was higher between 2010–2015 than 2002–2010 and indicate that grassland was the main land cover that replaced woody vegetation (forest and secondary vegetation) between 2010–2015.

When LULC trends are compared between political divisions during 2002–2010, we found that woody vegetation increased in the Colombian and Ecuadorian CGE during 2002–2010 (R = 0.78, p< 0.01; R = 0.64, p< 0.05) but did not show a significant trend in the Panamanian CGE. Secondary vegetation increased significantly in every national territory (R = 0.95, p< 0.01 for Panamá; R = 0.91 p< 0.01 for Colombia, and R = 0.85, p< 0.01 for Ecuador) while forest decreased (R = -0.89, p< 0.01 for Panamá; R = -0.95 p< 0.001 for Colombia, and R = -0.94, p< 0.01 for Ecuador). Grassland decreased in the Colombian CGE (R = -0.68 p< 0.04) but it did not show a significantly trend in Panamá and Ecuador. Crops decreased in the Ecuadorian CGE (R = -0.63 p< 0.05) and palm plantation decreased in the Colombian CGE (R = -0.86 p< 0.01). Between 2010–2015, some of the previous trends changed. Woody vegetation decreased significantly in Panamanian and Colombian CGE (R = -0.94, p< 0.01; R = -0.89, p< 0.02), forest maintained its decreasing trend in all three countries (R = -0.85, p< 0.02 for Panamá; R = -0.91 p< 0.01 for Colombia, and R = -0.96 p< 0.02 for Ecuador), grassland increased in Panamá and Colombia (R = 0.86, p< 0.03 for Panamá; R = 0.89 p< 0.02 for Colombia, and R = 0.63 p< 0.03 for Ecuador), and crops tended to decrease non significantly in the three countries (Table 1).

**Table 1:** Correlations of land-use and land-cover (LULC) change trends among administrative divisions; The Chocó-Darien Global Ecoregion (CGE), Colombian CGE (Col CGE), Ecuadorian CGE (Ecu CGE), and Panamanian CGE (Pan CGE). Pearson's correlation coefficient (R) are shown for two time periods 2002–2010 and 2010–2015. Significant codes: 0 ‘***’ 0.001 ‘**’ 0.01 ‘*’ 0.05.

Region	Time	Woody Veg. R(p)	Forest R(p)	Second. Veg. R(p)	Grass-land R(p)	Crops R(p)	Palm-Plan R(p)	Wet-land R(p)
CGE	2002–2010 2010–2015	0.81 (0.02)* -0.69 (0.13)	-0.96 (0.001)*** -0.98 (0.001)***	0.94 (0.01)** 0.17 (0.75)	-0.64 (0.05)* 0.85 (0.03)*	-0.65 (0.06) -0.75 (0.07)	-0.89 (0.02)* 0.89 (0.07)	0.51 (0.16) -0.56 (0.25)
Col CGE	2002–2010 2010–2015	0.78 (0.01)** -0.89(0.02)*	-0.95 (0.001)*** -0.91 (0.01)**	0.91 (0.01)** -0.14 (0.8)	-0.68 (0.04)* 0.89.(0.02)*	-0.33.(0.39) -0.57.(0.24)	-0.86.(0.01)** -0.01.(0.99)	0.08 (0.86) -0.54.(0.27)
Ecu CGE	2002–2010 2010–2015	0.64 (0.05)* -0.11 (0.84)	-0.94 (0.01)* * -0.96 (0.02)*	0.85 (0.01)** 0.22 (0.68)	0.14 (0.74) 0.03 (0.63)	-0.63 (0.05)* -0.72 (0.11)	-0.56 (0.15) 0.31 (0.54)	0.96 (0.01)** -0.14 (0.8)
Pan CGE	2002–2010 2010–2015	-0.57 (0.11) -0.94 (0.01)**	-0.89 (0.01)** -0.85 (0.02)*	0.95 (0.01)** 0.73 (0.1)	0.39 (0.3) 0.86 (0.03)*	0.06 (0.88) -0.78 (0.07)	0.1 (0.8) 0.36 (0.22)	0.36 (0.34) 0.75 (0.09)

**Fig 4:**
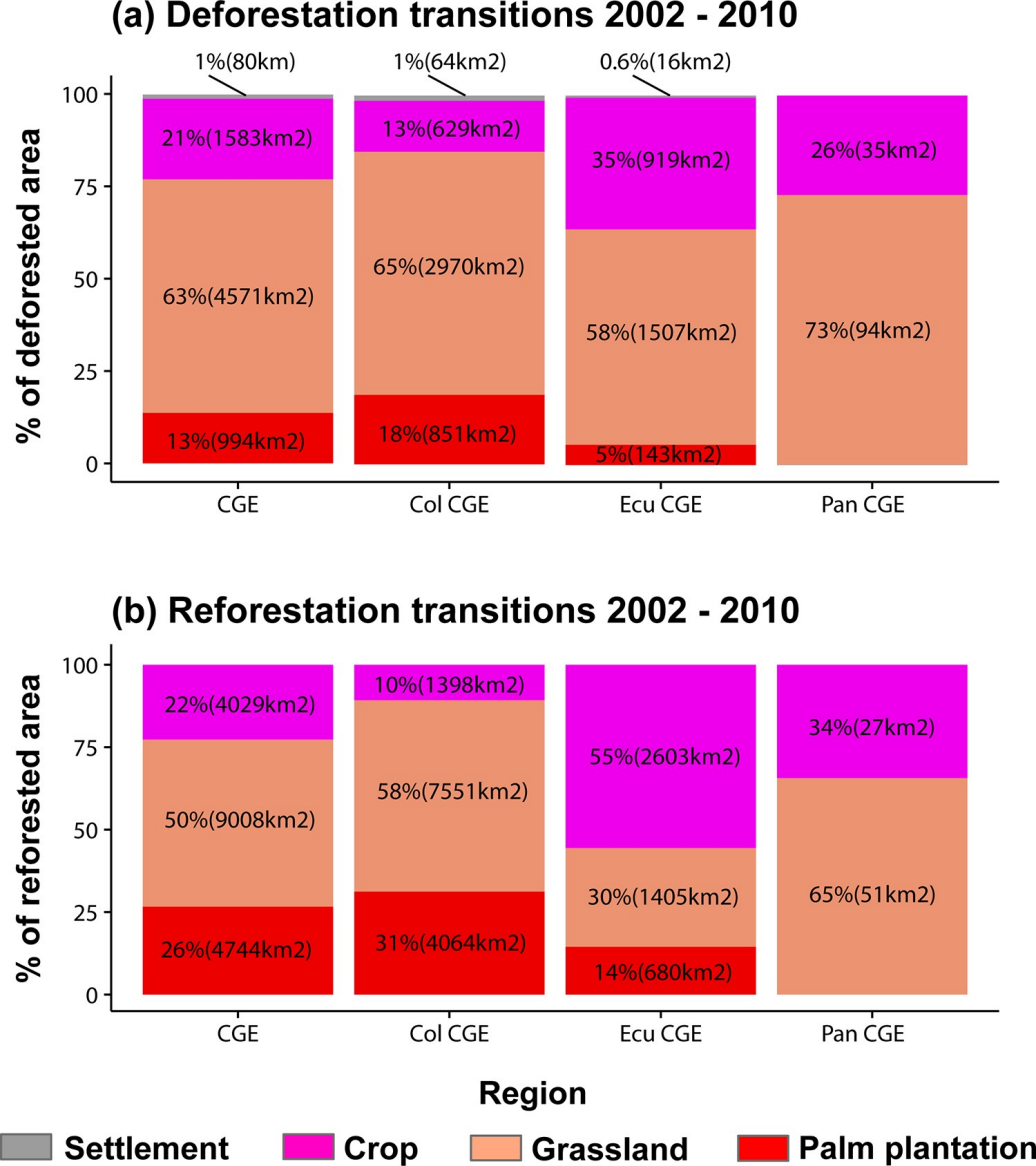
Quantification of deforestation (deforestation drivers) and reforestation transitions from 2002 to 2010. (a) Percentage of deforested area and net area deforested by every deforestation driver, and (b) percentage of reforested areas and net area reforested by every reforestation transition. The Chocó-Darien Global Ecoregion (CGE), Colombian CGE (Col CGE), Ecuadorian CGE (Ecu CGE), and Panamanian CGE (Pan CGE).

**Fig 5:**
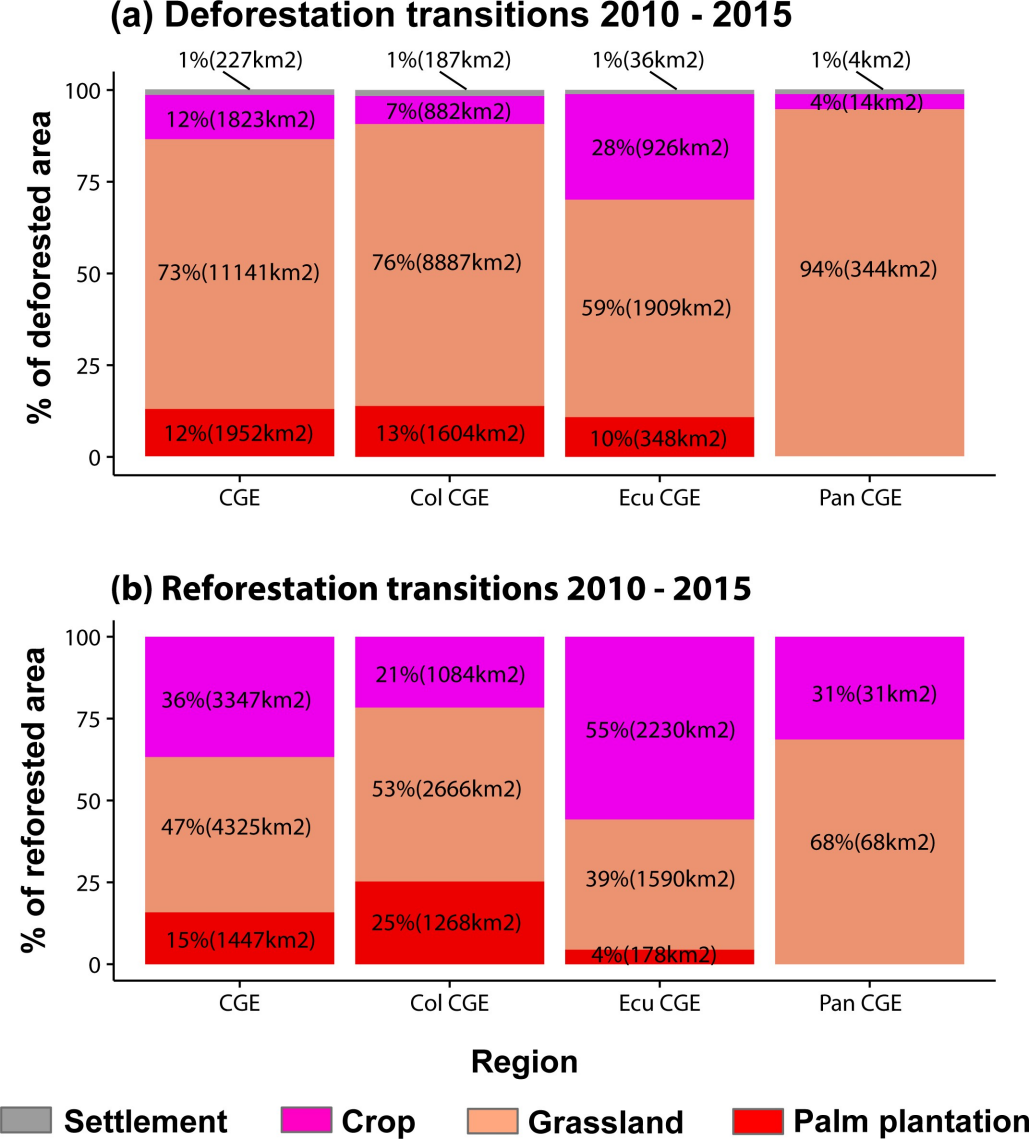
Quantification of deforestation (deforestation drivers) and reforestation transitions from 2010 to 2015. (a) Percentage of deforested area and net area deforested by every deforestation driver, and (b) percentage of reforested areas and net area reforested by every reforestation transition. The Chocó-Darien Global Ecoregion (CGE), Colombian CGE (Col CGE), Ecuadorian CGE (Ecu CGE), and Panamanian CGE (Pan CGE).

The analysis of LULC transition showed that grassland was the most frequent deforestation driver between 2002 to 2010 for the entire CGE (63%) and for each country (73% in Panamá, 65% in Colombia, and 58% in Ecuador) (Fig 4A; S6 Table). Grassland was also the most frequent land cover that change to secondary forest (reforestation) across the entire CGE (50%), in Panamá (65%), and Colombia (58%), but it was different in Ecuador where crops were the most frequent type to convert to secondary vegetation (55%) (Fig 4B; S6 Table). Subsequently, from 2010 to 2015, LULC transitions also showed that grassland was most frequent deforestation driver across the CGE (73%) as well as in every country (94% in Panamá, 76% in Colombia, and 59% in Ecuador) (Fig 5A; S7 Table). Grassland was also the most frequent land cover that converted to secondary vegetation during 2010–2015 for the CGE (47%) and in two countries (68% to Panamá and 53% to Colombia). In Ecuador, crops to secondary vegetation was the highest reforestation transition (55%) again (Fig 5B, S7 Table). The net deforestation was almost two times higher during 2010–2015 (15,145 km^2^) than 2002–2010 (7,228 km^2^) in the CGE; this pattern was similar in every country (Figs 4A and 5A). Conversely, net reforestation was higher between 2002–2010 (17783 km^2^) than 2010–2015 (9120 km^2^) in the CGE. As well, reforestation tended to be higher in every country during 2002–2010 compared to 2010–2015 (Figs 4B and 5B).

## Discussion

### Heterogeneity of LULC temporal dynamics

LULC change trends have been temporally heterogeneous across the CGE. We identified an overall increase in woody vegetation driven mainly by an increase in secondary vegetation between 2002–2010, this increase, however, ceased between 2010–2015. Conversely, grassland showed an overall decrease between 2002–2010 and an overall increase between 2010–2015. These trend shifts were similar between the Colombian and Ecuadorian portions (92% of CGE land) and suggest that external drivers could affected LULC change across the CGE. During the first decade of this century (2000–2010), the Colombian and Ecuadorian agricultural sectors declined, thus reducing cultivated (grassland, crops, palm) area and allowing for the growth of secondary vegetation. The Colombian agriculture sector decreased 1.1% during this period [84–86] while the Ecuadorian agricultural sector decreased by 1.8%. This was remarkable in Ecuador because its agricultural sector had grown by 6.1% between 1990–2000 [87]. Increases in secondary vegetation were also found in several developing countries within Latin America during the first ten years of the present century [5]. Some scholars have claimed that the globalization of markets negatively impacted the agriculture sectors of these countries resulting in abandonment of farm land and eventual reforestation [88,89]. Subsequently, from 2010 to 2015, Colombia and Ecuador showed a remarkable acceleration in their economic growth due to the global increase in the price of mining products (specially, oil, coal, energy, and gold). This acceleration could have a positive impact on all sectors of their economies (improving transportation routes, infrastructure in general, market for farming products) intensifying the use of farming areas. In Colombia, gross domestic agricultural product grew from negative values in 2009 to 5.5% in 2014 [90] and two important routes that cross large areas of the Colombian CGE were built (the route Tumaco- Tuquerres in Nariño department and the route Virginia-Quibdó in Risaralda and Choco departments). These routes correspond to some of the deforestation that we identified in our maps. In Ecuador, gross domestic agricultural product grew 6% from 2009 to 2013 [91]. This increase in agricultural production should have had a negative effect on the regeneration of secondary vegetation thus increasing deforestation as our results indicate. Some authors have claimed that reforestation in the Colombian CGE territory during 2002–2010 occurred principally due to land abandonment caused by internal armed conflicts in Colombia [34]. However, we found the same pattern in Ecuador during the same period (2002–2010), a country with no armed conflict. The regrowth of secondary vegetation across farming areas was proportionally higher in the Ecuadorian CGE compared to the Colombian CGE. Additionally, we found that reforestation has decreased significantly between 2010–2015 in the Colombian CGE while the armed conflict was still occurring and this area had a strong presence of the two main guerrilla groups in Colombia. This evidence suggests that economic growth could have a greater influence on the balance of deforestation and reforestation compared to local phenomenon such as armed conflicts. The Panamanian economy is not based on agriculture (main sectors in Panamá are transportation, communication, market, services and banking) [92]. This could explain the flat trend for woody vegetation in the Panamanian CGE through 2002–2010; however, reductions in woody vegetation, secondary vegetation and forest also occurred in the Panamanian CGE during 2010–2015 indicating increased human land use driven by economic growth during this time period. Panamá had the highest economic growth in Latin America between 2000–2013 (7.2% on average) [92,93]. Only forest had an overall consistent temporal trend cross the CGE, and tended to decline during both time periods across the three countries. Our split of woody vegetation into secondary vegetation and forest allowed us identify this progressive replacement of well-preserved forest primarily by grassland and secondary vegetation. Forest reduction has been documented in the Colombian CGE [8] and in the Ecuadorian CGE [26] between 2001–2010 using Landsat data and discriminations between forest and secondary vegetation.

Agricultural expansion was the most frequent deforestation driver during both time periods across the CGE; 98% of deforestation due to agricultural conversion and 1% by the establishment of settlement and infrastructure. Our results agree with other reports showing agricultural expansion as the main deforestation driver in the tropics [94,95]. In addition, we analyzed sub-categories of agricultural deforestation drivers (grassland, crops, and palm plantations) and found that grassland conversion was the main cause of deforestation across the CGE during both time periods. Extensive cattle grazing is a main agricultural activity for the areas corresponding to Magdalena-Urabá Ecoregion (46% of the CGE land and this entire sub-ecoregion is in Colombia) and to Western Ecuador Ecoregion (17% of the CGE land and the entire sub-ecoregion is in Ecuador) (Fig 1B) [96–99]. Other causes that explain the gradual replacement of forest by grassland and secondary vegetation cross the CGE during both time periods (2002–2010 and 2010–15) are the colonial process and the land possession policies of Colombia, Ecuador and Panamá. Basically, colonists are required to prove they are using land in order to become landowners. The cheapest and fastest method to prove land use is to convert forest to grassland. However, many of these deforested areas are underutilized and they consequently revert to secondary vegetation. Evidence supporting this hypothesis has been documented by other scholars; Davalos et al. (2014) found that forest conversion to grassland in several areas of the Amazon within Colombia were not related to beef production. They concluded that colonists were removing forest to prove active land use, gain ownership of the property, and wait for land values to increase [30]. IGAC (2015) found that deforestation after colonization in areas with fragile soils, such as Choco-Darien ecoregion of the CGE (Fig 1B), resulted in 38% of soils becoming unproductive in Colombia [100]. Historically, land possession has been a main source of economic and political power in Colombia and Ecuador resulting in land conflicts [9,101]. Consequently, future pressure on forest areas across the CGE could increase since this area hosts the largest population of colonist in Panamá and Ecuador [9,102].

Reforestation transitions were also heterogeneous cross the CGE. Grassland to secondary vegetation was the highest reforestation transition cross the CGE; however, it was different in the Ecuadorian CGE (16% of the CGE land) where crop conversion to secondary vegetation was the highest reforestation transition during both time periods (2002–2010 and 2010–2015). Agriculture consisting of annual or semiannual crops (corn, plantain, coffee, rice) was the principal driver of reforestation in the Ecuadorian CGE during 1990 and 2000 [9]. Manabí, Esmeraldas (the south side), and Santo Domingo (the largest Ecuadorian provinces in the CGE) are provinces considered to specialized in crop production, but cattle has increased since 2000 in this region while crops have decreased; presently, about 50% of the land consists of cultivated grassland and 18% by crops [103,104] and are consistent with our results.

Some scholars have claimed that palm plantations were one of the main drivers of deforestation in the CGE [53,105–107]. Our results showed that palm plantation was the third most significant deforestation driver across the CGE and its effect on forest and woody vegetation was different in every country; palm was the second deforestation driver in Colombia and the third in Ecuador. Panamá did not have palm plantations and thus it was not a factor in that country. Also, the reduction of forest as a result of palm plantations is substantial lower than the reduction produced by grassland cross the CGE. The zones that we identified as areas with palm plantation in Colombia coincide with the municipalities identified as areas with palm plantations by the Colombian Federation of Palm Farmers [108]. Specifically, we found that palm plantations were concentrated in three areas: Near the Colombia-Ecuador border, around the Urabá gulf, and cross Magdalena valley. As well, we found that palm plantations are partially spread cross the Ecuadorian CRB, which agrees with Ecuadorian studies about palm distribution; the Ecuadorian CGE is the region with the most palm plantations in this country and these cultivated areas have doubled between 2000 and 2010 [109].

Mining for mineral resources has been a primary historical economic activity along the Pacific coast of Colombia within the CGE. Due to the increasing price of gold, silver and platinum in international markets between 2010 and 2015, mining has increased with little governmental control in the Colombian Choco-Darien. Miners cut down forest, turn the soil, and separate minerals from soil material using mercury with water from nearby rivers. Additionally, areas are deforested to build roads to transport machinery [110,111]. Frequently, this mining activity occurs in smaller areas than our MODIS pixels size (231.3 m^2^); consequently, the spatial scale of our analysis did not allow us to study this driver of deforestation. Furthermore, up-to-date maps of mining activities do not exist and high resolution imagery for this portion of the study area are consistently cloud covered. Recently, the Colombian government has been using aerial cameras to document illegal mining in specific areas of the CGE, however, these methodologies are not applicable for an analysis of the entire region. Illegal farming activity, predominantly coca (*Erythroxylum coca*), is commonly found in the Colombian side (Nariño Department) near the border with Ecuador [112]. These areas were coincident with one of the deforestation areas that we identify in our maps. Although, we cannot discriminate coca crops from other farming activities, the documented distribution of this crop is evidence of its significant influence as a deforestation driver within the CGE.

### On the conservation of biodiversity

Considering the high diversity and endemism of the CGE, the rapid reduction of forest is a primary concern for conservation activities. Currently the CGE still has significant reserves of original forest. FAO (2010) estimated that 64% of the global woody vegetation corresponded to forest regeneration following anthropogenic disturbances [113]. We estimate that 34% of woody vegetation in the CGE in 2015 corresponded to secondary vegetation, suggesting that the CGE has a higher proportion of well conserved forest (42% by our estimate) than other areas across the world. These areas support high levels of biodiversity making them important for conservation. Tropical rain forest areas across the CGE occupied 83312 km^2^ in 2015; therefore, the CGE contains the second largest mass of tropical rain forest in South America, after the Amazon Basin. However, the fast and gradual replacement of forest areas by secondary vegetation points to another main concern. The high levels diversity and endemism prior to deforestation in these forests cannot be recovered after reforestations. That is, secondary forests evolving from secondary vegetation will have decreased biodiversity and different species assemblages [114].

The most conserved forests in the CGE are located in Panamá and along the pacific coast of Colombia. Human colonization has been restricted in these areas by two main geographic barriers, the Andes Mountains in the east and the Pacific Ocean to the west. However, the deforestation line has moved forward in two primary locations: to the east of the Colombia-Panamá border (in the northeast of these well-preserved forests) and on the Colombia-Ecuadorian border (to the south of these well-preserved forests). Mitigating deforestation in these two areas is critical to the conservation of the CGE. The integration of our time series of maps (built using standard remote sensing products) and field data on essential biodiversity variables [38,40] could be used to estimate biodiversity change in the context of LULC change.

### Map-production methodology

The methodology applied in this work created accurate LULC maps in one of the cloudiest areas of the planet. This methodology can be also used to create LULC maps with higher spatial resolution in cloudy areas using other sensors with relatively high temporal resolution. During the first 15 years of the 21 century, MODIS was the only sensor with moderate spatial resolution that offered enough temporal resolution to apply our methodology to build LULC maps in the CGE. After 2015, other sensors with higher spatial resolution, such as Landsat-7, Landsat-8 and Sentinel-2, have started to produce data with high temporal grain (when coupled together) for every part of the Earth. Merging the data produced by these sensors effectively increases their individual temporal grain and could be used to generate yearly LULC maps that allow for the identification of forest successions in greater detail.

Developing annual maps of LULC across the CGE using satellite-based remote sensing instruments with higher spatial resolution than MODIS had not been successful. The United Nations Collaborative Program on reducing emissions from deforestation and forest degradation (REDD) in Colombia used available Landsat imagery to develop four forest/non-forest land cover maps for the years 2000, 2005, 2010 and 2012 [115,116]. Each of these maps were developed using Landsat mosaics consisting of 3–4 contiguous years of imagery resulting in 13% of the area with no-information due to cloud cover. Our approach, using MODIS, allowed us to develop annual maps from 2002 to 2015 and identify LULC trends with a finer temporal grain. However, the MODIS pixel size cannot detect land cover change smaller than the 250 m^2^ nominal pixel size which could affect our results. We therefore compared the published trends of the four Landsat forest/no-forest maps from the Colombian REDD project with our MODIS maps for the same time periods. This analysis showed similar forest change trends between the Landsat and MODIS products; forest cover change trends were negatively correlated in similar proportions in the Landsat and MODIS maps (Landsat: R = -0.99, p = 0.003; MODIS: R = -0.97, p = 0.02). We also compared the woody vegetation change (forest and secondary vegetation) of our 2002 and 2014 MODIS land cover maps with the global forest change (GFC) maps of Hansen et al. (2013), which estimated loss and gain of tree cover between 2000 and 2014. To make an accurate comparison, we clipped the area classified as forest in our initial 2002 LULC map along with the LULC change between 2002 and 2014. We extracted the corresponding area of tree cover, tree loss and tree gain between 2000–2014 from the GFC database. The GFC product did not distinguish between forest (old forest) and secondary vegetation (young forests) as we did. Therefore, we combined these two classes into simply “woody vegetation” for the comparison. Our MODIS-based maps detected 6.35% woody vegetation loss between 2002 and 2014 compared to 3.9% for the GFC product. This level of non-agreement can be explained by the differences in spatial and temporal resolution as well as the definition of map classes between the GFC Landsat-based maps and our MODIS-based maps. The GFC database consists of two global maps of tree cover percentage for 2000 and 2014. The GFC database does not record the dynamics of tree cover between these two dates; consequently, the GFC does not discriminate between younger and older tree cover. Further, the increased spatial resolution of the GFC product compared to MODIS allows forest transitions to be identified at a finer scale. Small areas of non-forest within a matrix of forest tended to be classified as secondary forest using MODIS whereas the GFC product seemed to identify these areas as non-forest. Consequently, our MODIS-based product seemed to overestimate deforestation as compared to the GFC database. However, this difference is mitigated by the inclusion of widespread palm plantations and wetlands as tree cover in the GFC product where we were able exclude them from our classification of forest. A direct comparison, therefore is difficult.

We used the GFW processed MODIS MOD13Q1 to build the LULC annual maps. The MOD13Q1 dataset is a 250m resolution 16-day composite product calibrated to reflectance using an atmospheric correction for aerosol gases, and a BRDF (Bidirectional Reflectance Distribution Function) adjustment [117,118]. MOD13Q1 adopts two cloud filters [36,119] and an aerosol quality filter. Recently, other MODIS products, such as MOD09 (MOD09Q1 and MOD09A1), have been developed with improved cloud filtering using the MAIAC algorithm (Multi-Angle Implementation of Atmospheric Correction) [120]. We chose to use the MOD13Q1 product over the MOD09 products after comparing annual time series of NDVIs of both products. We found that, overall, the pre-GFW NDVI temporal sequence of MOD13Q1 (original data) time series are less variable than the NDVI temporal sequence of MOD09Q1 within the CGE, and where pixels coincided temporally between the two products on the 16-day cycle, the calculated NDVI values were often identical between the two products. Consequently, the MOD13Q1 time series after GWF had significantly less variation (t = 5.54; p = 0.02), allowing for a better discrimination between land cover types. Additionally, MOD09Q1 consists of only the first two spectral MODIS bands (red and NIR) which would not provide an EVI calculation and the MOD09A1 product, which allows for a calculation of EVI, has a spatial resolution of 500m reducing our ability to discriminate between spatially adjacent land cover types. Therefore, for our purposes, we found the MOD13Q1 product superior to the MOD09 products.

## Conclusions

By analyzing annual land-use and land-cover (LULC) change dynamics in the Chocó-Darien Global Ecoregion (CGE), we found that LULC change varied temporally. Deforestation and reforestation occurred across the CGE; however, deforestation increased after 2010 showing an increased risk for CGE conservation. We detected a gradual replacement of forest areas by secondary vegetation and agriculture, mainly grassland, which would then transition to secondary vegetation. The increased loss of forest after 2010 should be an important concern for the preservation of CGE biodiversity because forests in this ecoregion have high levels of species richness and endemism which are difficult to recover through reforestation. In other words, secondary forests evolving from secondary vegetation would have decreased biodiversity and different species assemblages [114].

We also found spatial variations that need to be considered when developing CGE-wide management plans aimed at preserving biodiversity and ecosystem services. Across national boundaries, the Ecuadorian section had the smallest proportion of forest (11%; 3578.6 km^2^; mostly located in the north near the border with Colombia), for that reason, restoration programs are urgently needed in the Ecuadorian CGE. The Colombian CGE had the largest area of forest (66160 km^2^; mostly located in the east along the pacific coast from the Panamanian border, south to the northern border of the Cauca Department) but also the largest deforested area. The Panamanian CGE contains the largest proportion of forest within their boundaries (88%; 13569 km^2^) but this forested area is only 8% of the CGE. However, the forest in the Panamanian CGE are fundamental to the connection of fauna and flora between Central and South America because these forests span the Isthmus of Panamá a land bridge for the biodiversity for the American continent. Regions with high deforestation transitions, such as the southern Colombian CGE, show areas where forest protection strategies should be implemented. Whereas regions with high reforestation transitions can identify areas in which forest restoration programs might be established, as the north of the Ecuadorian CGE, for example.

Our methodological approach for producing accurate LULC maps can be applied in other cloudy regions using open source software and imagery available at no cost.

## Supporting information

S1 Table High spatial resolution imagery used to the visually interpreting of the land-use and land-cover (LULC) classes. (DOCX)

S2 Table Table of response and predictor variables used in the Random Forest classification. S2 Table is in CSV format. This table can be download in the next link https://zenodo.org/record/2543865#.XEI9llxKiM8.
(DOCX)

S3 Table Original distribution of the land-use and land-cover (LULC) classes and sampling reduction. (DOCX)

S4 Table Confusion matrix of the second independent group from the original data. Kappa, commissions and omissions are in the matrix.
(DOCX)

S5 Table Confusion matrix of the second independent group from the data when woody vegetation class is reduced as the grassland class number. Kappa, commission and omission are in the matrix.
(DOCX)

S6 Table Deforestation (deforestation drivers) and reforestation transitions from 2002 to 2010. (DOCX)

S7 Table Deforestation (deforestation drivers) and reforestation transitions from 2010 to 2015. (DOCX)

S1 Codes R codes used for the Random Forest classification (run with S2 Table). (DOCX)
