# Correction: Geospatial modeling of land cover change in the Chocó-Darien global ecoregion of South America; One of most biodiverse and rainy areas in the world

**DOI:** 10.1371/journal.pone.0213315

**Published:** 2019-02-27

**Authors:** The PLOS ONE Staff

## Notice of republication

This article was republished on February 15, 2019 to correct for errors in Fig 3 introduced during the typesetting process. The publisher apologizes for the errors. Please download this article again to view the correct version. The originally published, uncorrected article and the republished, corrected article are provided here for reference.

## Supporting information

S1 File Originally published, uncorrected article. (PDF)

S2 File Republished, corrected article. (PDF)
