# Supplementary material for: Geospatial modeling of land cover change in the Chocó-Darien global ecoregion of South America; One of most biodiverse and rainy areas in the world
Source: PLoS One. 2019 Feb 1;14(2):e0211324. doi: 10.1371/journal.pone.0211324 (PMC6358088; doi:10.1371/journal.pone.0211324)
Supplement: S1 Table — (DOCX) [file pone.0211324.s001.docx]

S1 Table. High spatial resolution imagery used to the visually interpreting of the land-use and land-cover (LULC) classes.

| Sensor | Band Resolution | Bands | Pancromatic Resolution | Cover  (Km^2^) | Year | Image name |
| --- | --- | --- | --- | --- | --- | --- |
| GeoEye-1 | 2.2 | 4 | 0.5 | 1000 | 2012 | 12SEP03155046-P2AS-053711282080 |
| GeoEye-1 | 2.2 | 4 | 0.5 | 2212 | 2011 | 11MAR12153945-M2AS-053711282090 |
| GeoEye-1 | 2.2 | 4 | 0.5 | 1042 | 2012 | 12APR05154853-M2AS-054758991030 |
| GeoEye-1 | 2.2 | 4 | 0.5 | 1915 | 2012 | 12APR02153914-P2AS-053738769050 |
| Ikonos | 3.2 | 4 | 0.8 | 568 | 2006 | po1473219 |
| Ikonos | 3.2 | 4 | 0.8 | 189 | 2005 | po_1472872 |
| QuickBird | 2.4 | 4 | 0.6 | 516 | 2007 | 07AUG01154941-M2AS-0546 |
| QuickBird | 2.4 | 4 | 0.6 | 1075 | 2012 | 12JUN29145741-M2AS-054635510010 |
| QuickBird | 2.4 | 4 | 0.6 | 526 | 2012 | 12FEB17145933-M2AS-053738769040 |
| QuickBird | 2.4 | 4 | 0.6 | 400 | 2002 | 02JUN12154300-M2AS-053738769030 |
| QuickBird | 2.4 | 4 | 0.6 | 633 | 2003 | 03FEB01154326-M2AS-053711282070 |
| QuickBird | 2.4 | 4 | 0.6 | 172 | 2010 | 10SEP101001000B90ED00-MS1 |
| QuickBird | 2.4 | 4 | 0.6 | 38 | 2011 | Panamá_net_point_463.csv |
| QuickBird | 2.4 | 4 | 0.6 | 170 | 2011 | Panamá2_net_point_463.csv |
| QuickBird | 2.4 | 4 | 0.6 | 725 | 2011 | 12SEP101001000E351600-MS1 |
| QuickBird | 2.4 | 4 | 0.6 | 54 | 2012 | 12DEC1010010010CD4300 |
| WORLDVIEW-2 | 2.2 | 8 | 0.5 | 2526 | 2011 | 11DEC103001000F0FD300-MS2 |
| WORLDVIEW-2 | 2.2 | 8 | 0.5 | 222 | 2011 | 11FEB14155742-M2AS-054758991010 |
| WORLDVIEW-2 | 2.2 | 8 | 0.5 | 270 | 2015 | 15APR04154546-M2AS-054758991020 |
| WORLDVIEW-2 | 2.2 | 8 | 0.5 | 2018 | 2013 | 13JUL09154910-M2AS-054635510030 |
| WORLDVIEW-2 | 2.2 | 8 | 0.5 | 2106 | 2013 | 13DEC02161527-M2AS-053738769020 |
| WORLDVIEW-2 | 2.2 | 8 | 0.5 | 2320 | 2011 | 11OCT23161008-M2AS-053738769010 |
| Area |  |  |  | 20708 |  |  |
