# Supplementary material for: Geospatial modeling of land cover change in the Chocó-Darien global ecoregion of South America; One of most biodiverse and rainy areas in the world
Source: PLoS One. 2019 Feb 1;14(2):e0211324. doi: 10.1371/journal.pone.0211324 (PMC6358088; doi:10.1371/journal.pone.0211324)
Supplement: S2 Table — is in CSV format. This table can be download in the next link https://zenodo.org/record/2543865#.XEI9llxKiM8. (DOCX) [file pone.0211324.s002.docx]

S2 Table. Table of response and predictor variables used in the Random Forest classification. S2 Table is in CSV format. This table can be download in the next link <https://zenodo.org/record/2543865#.XEI9llxKiM8>
