# Supplementary material for: Geospatial modeling of land cover change in the Chocó-Darien global ecoregion of South America; One of most biodiverse and rainy areas in the world
Source: PLoS One. 2019 Feb 1;14(2):e0211324. doi: 10.1371/journal.pone.0211324 (PMC6358088; doi:10.1371/journal.pone.0211324)
Supplement: S3 Table — (DOCX) [file pone.0211324.s003.docx]

S3 Table. Original distribution of the land-use and land-cover (LULC) classes and sampling reduction.

| Land cover | Class distribution | Class distribution after Woody vegetation reduction |
| --- | --- | --- |
| Woody vegetation | 14228 | 1144 |
| Grassland | 1144 | 1144 |
| Crop | 404 | 404 |
| Palm | 743 | 743 |
| Urban | 121 | 121 |
| Water | 1123 | 1123 |
| Wetland | 796 | 796 |
