# Supplementary material for: Geospatial modeling of land cover change in the Chocó-Darien global ecoregion of South America; One of most biodiverse and rainy areas in the world
Source: PLoS One. 2019 Feb 1;14(2):e0211324. doi: 10.1371/journal.pone.0211324 (PMC6358088; doi:10.1371/journal.pone.0211324)
Supplement: S4 Table — Kappa, commissions and omissions are in the matrix. (DOCX) [file pone.0211324.s004.docx]

S4 Table. Confusion matrix of the second independent group from the original data. Kappa, commissions and omissions are in the matrix.

| Kappa | Kappa.sd | Observed | | | | | | | | |
| --- | --- | --- | --- | --- | --- | --- | --- | --- | --- | --- |
| 0.876 | 0.008 | Woody Vegetation | Grassland | Crop | Palm | Urban | Water | Wetland | total | Commission |
| Predicted | Woody vegetation | 2833 | 39 | 9 | 20 | 1 | 1 | 15 | 2918 | 0.03 |
|  | Grassland | 14 | 151 | 11 | 5 | 0 | 0 | 1 | 182 | 0.17 |
|  | Crop | 2 | 6 | 42 | 10 | 0 | 0 | 1 | 61 | 0.31 |
|  | Palm | 11 | 6 | 1 | 108 | 0 | 0 | 0 | 126 | 0.14 |
|  | Urban | 0 | 0 | 0 | 0 | 20 | 0 | 0 | 20 | 0.00 |
|  | Water | 0 | 2 | 0 | 0 | 0 | 229 | 2 | 233 | 0.02 |
|  | Wetland | 12 | 1 | 2 | 1 | 0 | 2 | 154 | 172 | 0.10 |
|  | total | 2872 | 205 | 65 | 144 | 21 | 232 | 173 | 3712 | PCC |
|  | Omission | 0.01 | 0.26 | 0.35 | 0.25 | 0.05 | 0.01 | 0.11 | PCC | 0.95 |
| MAUC | 0.983 | cmx | cmx | cmx | cmx | cmx | cmx | cmx | cmx | cmx |
