# Supplementary material for: Geospatial modeling of land cover change in the Chocó-Darien global ecoregion of South America; One of most biodiverse and rainy areas in the world
Source: PLoS One. 2019 Feb 1;14(2):e0211324. doi: 10.1371/journal.pone.0211324 (PMC6358088; doi:10.1371/journal.pone.0211324)
Supplement: S5 Table — Kappa, commission and omission are in the matrix. (DOCX) [file pone.0211324.s005.docx]

S5 Table. Confusion matrix of the second independent group from the data when woody vegetation class is reduced as the grassland class number. Kappa, commission and omission are in the matrix.

| Kappa | Kappa.sd | Observed | | | | | | | | | |
| --- | --- | --- | --- | --- | --- | --- | --- | --- | --- | --- | --- |
| 0.873 | 0.01 | Woody Vegetation | Grassland | Crop | Palm | Urban | Water | Wetland | Total | Commission |  |
| Predicted | Woody vegetation | 185 | 7 | 5 | 1 | 0 | 1 | 2 | 201 | 0.08 |  |
|  | Grassland | 7 | 200 | 17 | 7 | 1 | 0 | 6 | 238 | 0.16 |  |
|  | Crop | 1 | 11 | 57 | 14 | 0 | 0 | 1 | 84 | 0.32 |  |
|  | Palm | 6 | 4 | 3 | 125 | 0 | 0 | 2 | 140 | 0.11 |  |
|  | Urban | 0 | 1 | 0 | 0 | 22 | 2 | 0 | 25 | 0.12 |  |
|  | Water | 0 | 1 | 0 | 0 | 0 | 224 | 1 | 226 | 0.01 |  |
|  | Wetland | 3 | 5 | 1 | 1 | 0 | 3 | 156 | 169 | 0.08 |  |
|  | Total | 202 | 229 | 83 | 148 | 23 | 230 | 168 | 1083 | PCC |  |
|  | Omission | 0.08 | 0.13 | 0.31 | 0.16 | 0.04 | 0.03 | 0.07 | PCC | 0.89 |  |
| MAUC | 0.985 | cmx | cmx | cmx | cmx | cmx | cmx | cmx | cmx | cmx |  |
