# Supplementary material for: Geospatial modeling of land cover change in the Chocó-Darien global ecoregion of South America; One of most biodiverse and rainy areas in the world
Source: PLoS One. 2019 Feb 1;14(2):e0211324. doi: 10.1371/journal.pone.0211324 (PMC6358088; doi:10.1371/journal.pone.0211324)
Supplement: S6 Table — (DOCX) [file pone.0211324.s006.docx]

S6 Table. Deforestation (deforestation drivers) and reforestation transitions from 2002 to 2010.

|  | Deforested | | | |  | Reforested | | | |
| --- | --- | --- | --- | --- | --- | --- | --- | --- | --- |
|  | CGE | Col | Ecu | Pan |  | CGE | Col | Ecu | Pan |
| Grassland |  |  |  |  |  |  |  |  |  |
| Area (km2) | 4571 | 2970 | 1507 | 94 |  | 9008 | 7551 | 1405 | 51 |
| Prop. of total (%) | (63.2) | (65.8) | (58.3) | (73) |  | (50.7) | (58) | (30) | (65.6) |
|  |  |  |  |  |  |  |  |  |  |
| Crop |  |  |  |  |  |  |  |  |  |
| Area (km^2^) | 1583 | 629 | 919 | 35 |  | 4029 | 1398 | 2603 | 27 |
| Prop. of total (%) | (21.9) | (13.9) | (35.6) | (27) |  | (22.7) | (10.7) | (55.5) | (34.4) |
|  |  |  |  |  |  |  |  |  |  |
| Palm |  |  |  |  |  |  |  |  |  |
| Area (km^2^) | 994 | 851 | 143 | 0 |  | 4744 | 4064 | 680 | 0 |
| Prop. of total (%) | (13.7) | (18.8) | (5.5) | (0) |  | (26.7) | (31.2) | (14.5) | (0) |
|  |  |  |  |  |  |  |  |  |  |
| Settlement |  |  |  |  |  |  |  |  |  |
| Area (km^2^) | 80 | 64 | 16 | 0 |  | 0 | 0 | 0 | 0 |
| Prop. of total (%) | (1.1) | (1.4) | (0.6) | (0.1) |  | (0) | (0) | (0) | (0) |
|  |  |  |  |  |  |  |  |  |  |
| Total |  |  |  |  |  |  |  |  |  |
| Area (km^2^) | 7228 | 4514 | 2585 | 129 |  | 17783 | 13014 | 4689 | 79 |
| Prop. of total (%) | (100) | (100) | (100) | (100) |  | (100) | (100) | (100) | (100) |
|  |  |  |  |  |  |  |  |  |  |
