# Supplementary material for: Geospatial modeling of land cover change in the Chocó-Darien global ecoregion of South America; One of most biodiverse and rainy areas in the world
Source: PLoS One. 2019 Feb 1;14(2):e0211324. doi: 10.1371/journal.pone.0211324 (PMC6358088; doi:10.1371/journal.pone.0211324)
Supplement: S7 Table — (DOCX) [file pone.0211324.s007.docx]

S7 Table. Deforestation (deforestation drivers) and reforestation transitions from 2010 to 2015.

|  | Deforested | | | |  | Reforested | | | |
| --- | --- | --- | --- | --- | --- | --- | --- | --- | --- |
|  | CGE | Col | Ecu | Pan |  | CGE | Col | Ecu | Pan |
| Grassland |  |  |  |  |  |  |  |  |  |
| Area (km^2^) | 11141 | 8888 | 1909 | 344 |  | 4325 | 2667 | 1590 | 68 |
| Prop. of total (%) | (73.6) | (77) | (59.3) | (94.6) |  | (47.4) | (53.1) | (39.8) | (68.7) |
|  |  |  |  |  |  |  |  |  |  |
| Crop |  |  |  |  |  |  |  |  |  |
| Area (km^2^) | 1823 | 882 | 926 | 14 |  | 3347 | 1084 | 2231 | 31 |
| Prop. of total (%) | (12) | (7.6) | (28.8) | (4.1) |  | (36.7) | (21.6) | (55.8) | (31.3) |
|  |  |  |  |  |  |  |  |  |  |
| Palm |  |  |  |  |  |  |  |  |  |
| Area (km^2^) | 1953 | 1604 | 348 | 0 |  | 1447 | 1268 | 178 | 0 |
| Prop. of total (%) | (13) | (14) | (10.8) | (0) |  | (15.9) | (25.3) | (4.5) | (0) |
|  |  |  |  |  |  |  |  |  |  |
| Settlement |  |  |  |  |  |  |  |  |  |
| Area (km^2^) | 228 | 187 | 36 | 4 |  | 0 | 0 | 0 | 0 |
| Prop. of total (%) | (1.5) | (1.6) | (1.1) | (1.3) |  | (0) | (0) | (0) | (0) |
|  |  |  |  |  |  |  |  |  |  |
| Total |  |  |  |  |  |  |  |  |  |
| Area (km^2^) | 15145 | 11561 | 3220 | 363 |  | 9120 | 5020 | 4000 | 99 |
| Prop. of total (%) | (100) | (100) | (100) | (100) |  | (100) | (100) | (100) | (100) |
|  |  |  |  |  |  |  |  |  |  |
