# Supplementary material for: Geospatial modeling of land cover change in the Chocó-Darien global ecoregion of South America; One of most biodiverse and rainy areas in the world
Source: PLoS One. 2019 Feb 1;14(2):e0211324. doi: 10.1371/journal.pone.0211324 (PMC6358088; doi:10.1371/journal.pone.0211324)
Supplement: S1 Codes — (DOCX) [file pone.0211324.s008.docx]

S1 Codes. R codes used for the Random Forest classification (run with S2 Table).

################################## Random Forest classification

library(randomForest)

library(ModelMap)

model.type <- "RF"

qdatafn <- "LA_TABLA S2.csv"

qdata.trainfn <- "VModelMapData_TRAIN_cathegorical.csv"

qdata.testfn <- "VModelMapData_TEST_cathegorical.csv"

folder <- getwd()

get.test( proportion.test=0.2, #Percentage for the cross validation

qdatafn=qdatafn,

seed=42,

folder=folder,

qdata.trainfn=qdata.trainfn,

qdata.testfn=qdata.testfn)

MODELfn <- "VModelMapEx3"

predList <- c( "evi",

"mir",

"ndvi",

"nir",

"red",

"slope",

"altitud"

)

response.name <- "cover"

response.type <- "categorical"

seed <- 44

unique.rowname <- "ID"

##########To build maps (Use only if you have the raster predictors)

#rastLUTfn <- "VModelMapData_LUT.csv"

#rastLUTfn <- read.table( rastLUTfn,

#header=FALSE,

#sep=",",

#stringsAsFactors=FALSE)

#rastLUTfn[,1] <- paste(folder,rastLUTfn[,1],sep="/")

################################## Creation of the model

model.obj.ex3 <- model.build( model.type=model.type,

qdata.trainfn=qdata.trainfn,

folder=folder,

unique.rowname=unique.rowname,

MODELfn=MODELfn,

predList=predList,

predFactor=FALSE,

response.name=response.name,

response.type=response.type,

seed=seed)

################################## Model diagnosis

model.pred.ex3 <- model.diagnostics( model.obj=model.obj.ex3,

qdata.testfn=qdata.testfn,

folder=folder,

MODELfn=MODELfn,

unique.rowname=unique.rowname,

prediction.type="TEST", #By type TEST, the validation predictions

#will be made on the test set provided by qdata.testfn.

# or by type "OOB" you get the kappa of the out-of-bag (OOB) validations

device.type="jpeg",

cex=1.2)

################################## Commands for doing the map (Use only if you have the raster predictors)

#model.mapmake( model.obj=model.obj.ex3,

#folder=folder,

#MODELfn=MODELfn,

#rastLUTfn=rastLUTfn,

#na.action="na.omit")

################################## To allocate codes to my the land covers (Use only if you have the raster predictors)

#MAP.CODES<-read.table( paste(MODELfn,"_map_key.csv",sep=""),

#header=TRUE,

#sep=",",

#stringsAsFactors=FALSE)

#MAP.CODES

#write.csv(MAP.CODES, file = "MAP_CODES")

################################## To allocate codes to my the land covers (Use only if you have the raster predictors)
